# The changing epidemiology of malaria in Minnesota.

**DOI:** 10.3201/eid0706.010612

**Published:** 2001

**Authors:** S A Seys, J B Bender

**Affiliations:** Acute Dieases Epidemiology Section, Minnesota Department of Public Health, Minneapolis, USA. saseys@state.wy.us

## Abstract

Malaria cases reported to the Minnesota Department of Health increased from 5 in 1988 to 76 in 1998, paralleling the number of immigrants to Minnesota. In 20% of cases, the Plasmodium species was not identified; 44% of cases were hospitalized. The public health community needs to reevaluate current recommendations for refugee screening, provider and patient education, and laboratory capacity.


### The Changing Epidemiology of Malaria in Minnesota

**Scott A. Seys and Jeff B. Bender**
Minnesota Department of Health, Minneapolis, Minnesota, USA

Malaria cases reported to the Minnesota Department of Health increased from 5 in 1988 to 76 in 1998, paralleling the number of immigrants to Minnesota. In 20% of cases, the *Plasmodium* species was not identified; 44% of cases were hospitalized. The public health community needs to reevaluate current recommendations for refugee screening, provider and patient education, and laboratory capacity.

Malaria infects 300 to 500 million people worldwide and accounts for over 1 million deaths annually (1). Of all infectious diseases, it is second only to tuberculosis in the number of people killed (1). As a result of political unrest and economic hardship, many refugees and immigrants from malaria-endemic areas are moving to nonendemic countries. This provides unique challenges to health-care providers, who may be confronted with diseases not previously observed during their training or clinical practice.

This study examined the changing epidemiology of imported malaria, i.e., malaria that is acquired abroad but diagnosed in the United States. Our goal was to add to previous knowledge of imported malaria by summarizing surveillance data from 1988 through 1998 and by discussing the implications for refugee screening, provider and patient education, and laboratory capacity.

### The Study

Malaria is a reportable disease to the Minnesota Department of Health (MDH) and the Centers for Disease Control and Prevention. All cases of malaria reported from January 1988 to December 1998 were included in this study. A confirmed case was one diagnosed by microscopy in a Minnesota resident with *Plasmodium* species. When a case was reported, a standardized malaria case surveillance form was completed by telephone in consultation with medical providers, laboratory staff, and the patient. Data collected include demographic information, clinical history, travel and immigration history, history of prior malarial infections, and species of *Plasmodium*.

Cases of malaria were categorized as follows: cases in travelers or immigrants, U.S. born or foreign born persons, and U.S. citizen or non-U.S. citizen. A traveler was defined as a person whose travel originated and ended in Minnesota; an immigrant was defined as a person whose travel originated in a foreign country. Citizenship was based on the reported status at the time of diagnosis.

From January 1, 1988, to December 31, 1998, 265 cases of malaria were reported to the MDH. The number of cases per year ranged from a low of 5 cases in 1988 to 76 cases in 1998 (Figure). Demographic characteristics of cases are presented in the Table. Of the 212 cases with reported travel status, 138 (65%) were considered travelers from Minnesota, and 74 (35%) cases were immigrants to Minnesota. From 1988 through 1994, the percentage of cases in travelers was as high as 94%. In 1995 the ratio of travelers to immigrants began to change. By 1998 there were 38 (54%) immigrants and 32 (46%) travelers in the 70 cases with a known status (chi square for linear trend = 15.0; p<0.005).

Among those with known citizenship status (n=164), U.S. citizens accounted for up to 78% of the cases per year in the period from 1988 through 1994. By 1998, 18 (28%) of 65 malaria cases (11 cases had an unknown status) had U.S. citizenship, while 47 (72%) were non-U.S. citizens (chi square test for linear trend = 13.5; p<0.005).

In 1998, excluding 10 cases for which we could not ascertain birthplace, 13 (20%) cases were born in the United States and 53 (80%) abroad. Of those born abroad with a known country of birth, 33 (87%) were born in Africa, 20 (53%) of these in Liberia (West Africa). Among the 8 patients who were born abroad, sites of malarial infection were Africa and Asia. Most patients typically traveled to or originated as immigrants from West-Central Africa or the Greater Horn of Africa. Liberia (n=29, 55%), the Ivory Coast (n=9, 17%), Kenya (n=6, 11%), Ethiopia (n=4, 8%), and Nigeria (n=4, 8%) were the most common countries where exposure to malaria likely occurred. For the 13 patients in 1998 who were born in

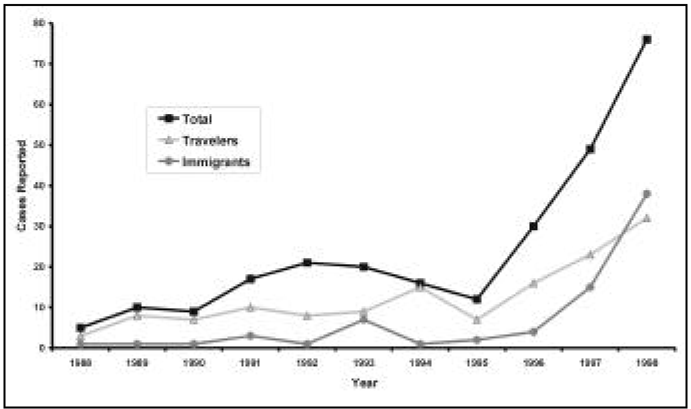

Figure. Reported cases of malaria by year, Minnesota, 1988-1998.

Address correspondence to: Scott Seys, Epidemiology Section, Wyoming Department of Health, Hathaway Building-Fourth Floor, 2300 Capitol Avenue, Cheyenne, Wyoming, 82002, USA; fax: 307-777-5573; e-mail: saseys@state.wy.us

Table. Demographic characteristics of malaria cases, Minnesota, 1988-1998 (n=265)

| Characteristic | No. of respondents | n | %[a] |
|---|---|---|---|
| Sex | 265 | | |
| Male | | 180 | 67.9 |
| Female | | 85 | 32.1 |
| Residence | 264 | | |
| Twin Cities (seven-county metropolitan area) | | 211 | 79.9 |
| Hennepin County | | 129 | 48.9 |
| Ramsey County | | 63 | 23.9 |
| Age (years) | 260 | | |
| ≤5 | | 26 | 10.0 |
| 6-17 | | 44 | 16.9 |
| 18-29 | | 66 | 25.4 |
| 30-44 | | 76 | 29.2 |
| 45-64 | | 43 | 16.5 |
| ≥65 | | 5 | 1.9 |
| Race | 217 | | |
| Black | | 130 | 59.9 |
| White | | 58 | 26.7 |
| Asian/Pacific Islander | | 23 | 10.6 |
| Hispanic | | 5 | 2.3 |
| American Indian | | 1 | 0.5 |
| Travel origin | 212 | | |
| Traveled from Minnesota | | 138 | 65.1 |
| Immigrated to Minnesota | | 74 | 34.9 |
| Citizenship | 164 | | |
| Non-U.S. citizen | | 95 | 57.9 |
| Primary refugee | | 37 | 22.6 |
| U.S. citizen | | 69 | 42.1 |

[a]Calculations are based on the number of respondents.

the United States, infection occurred in Africa (n=6, 46%), Asia (n=3, 23%), Central America (n=1, 8%), and South America (n=1, 8%). Two patients (15%) were potentially exposed in more than one continent.

Most cases since 1988 were diagnosed with *Plasmodium falciparum* (n=111; 42%), followed by *P. vivax* (n=76; 29%), *P. malariae* (n=14; 5%), and *P. ovale* (n=4; 2%). Laboratory studies of eight cases (3%) showed a mixed infection. *Plasmodium* was identified but no species was determined for 52 (20%) cases. In 1998, there was only 1 U.S.-born case (11%) of *P. falciparum* compared with 25 foreign-born cases (64%). The proportion of U.S.-born cases with *P. vivax* in 1998 was greater than foreign-born cases (odds ratio [OR]=undefined; Fisher's exact 2-tailed test, $p<0.005$). There were two cases of *P. malariae* in 1998; both were in immigrants born in Liberia.

Eleven foreign-born cases from 1998 were asymptomatic and were screened as part of one hospital's refugee assessment. In the period from 1988 through 1998, 109 (44%) of 245 cases were hospitalized for 1 to 30 days (median=3 days). Data from 1998 showed no significant difference in rates of hospitalization between those born in the United States and those born abroad. Complications were reported for 19 (11%) of 170 cases from 1988 through 1998. Data were not available on 95 cases. Two patients were diagnosed with cerebral malaria, 18 with hemolysis or anemia, and 1 with liver failure. No deaths were reported during the 11-year period. No cases of locally acquired or blood transfusion-associated malaria were reported to MDH in 1988 through 1998.

Ninety-nine (52%) of 189 patients indicated that they had a previous history of malaria. In 1998, 37 (59%) of 63 cases had been previously diagnosed with malaria. Patients born outside the United States accounted for 30 (97%) of these malaria cases with a known country of birth.

### Conclusions

We reviewed 265 malaria cases from 1988 through 1998 reported to the MDH. We found that cases, especially among refugees and immigrants, had increased; 20% of cases did not have malarial species identification from blood smears; and 44% were hospitalized. These findings have an impact on current recommendations for refugee screening, provider and patient education, and appropriate laboratory capacity to determine malarial species.

Before the twentieth century, much of the Midwest was endemic for malaria (2). In Minnesota, the last reported cases of locally acquired malaria most likely occurred in the 1930s (3). Since then, there have been major peaks of reported malaria diagnosed in Minnesota that have coincided with the return of soldiers from wars or immigration.

The dramatic climb in cases diagnosed and reported to the MDH in 1997 and 1998 and the increasing proportion of cases among immigrants correspond with increases in primary refugees from Liberia. The number of primary refugees from Liberia climbed from 18 in 1996 to 122 in 1997 and then to 205 in 1998 (MDH, unpub. data). Minnesota was second only to New York in the number of Liberian refugees resettling in the state in fiscal year 1998 (4).

Clinical laboratory training and availability of tests to identify *Plasmodium* at the species level are needed. Species had not been determined for 20% of cases reported to MDH. This has an impact on treatment recommendations because drug resistance and treatment are dependent not only on the country of acquisition but also the species of *Plasmodium*. Diagnosis of malaria is most frequently done by parasite identification on peripheral blood smears. Many laboratory diagnosticians may be uncomfortable diagnosing to the species level (5). Available polymerase chain reaction methods can diagnose malaria at the species level but are limited to reference laboratories (6).

In this study, more than one of every three cases was hospitalized for 1 to 30 days. This may have an impact financially and culturally on immigrants and refugees who have recently arrived in the United States. Many new arrivals may be uninsured or underinsured and have limited or no prior exposure to western medicine.

In 1998, we noted that 11 (14%) of 76 cases were asymptomatic; all were foreign-born immigrants, and 10 (91%) were primary refugees to Minnesota. In Minnesota, initial health screening for infectious diseases in primary refugees includes tuberculosis, sexually transmitted diseases, hepatitis B, intestinal parasites, and malaria if the person is symptomatic (7). We propose that all primary refugees from malaria-endemic areas be screened for malaria whether or not the person is symptomatic. This would likely prevent future health problems in refugee populations and reduce the risk of autochthonous malaria transmission.

National efforts are needed to support refugee and immigrant health programs that improve access to health care for these populations. Education for health-care providers is needed so that they screen immigrants and refugees appropriately and provide relevant pre-travel advice to those planning return visits to their country of origin. Also needed are culturally sensitive materials for refugees and immigrants that are written in their primary language. This presents unique challenges considering that the diversity of populations resettling in the United States will continue to change, depending on the location of current political and social unrest. The public health community needs to consider these important issues and recommendations as we continue to monitor the influence of immigration on the changing epidemiology of malaria.

### Acknowledgments

We thank Kristin Anderson and DeAnn Lazovich for their guidance on the early version of this manuscript; Kaying Hang, Ann O'Fallon, and Dzung Thai for their helpful information and support; and Richard Danila, Alan Lifson, Dave Neitzel, and Kirk Smith for helping to review this manuscript and for sharing their knowledge and expertise.

Mr. Seys is currently the infectious disease epidemiologist and chief of the Epidemiology Section at the Wyoming Department of Health. At the time of this research, he worked in the Acute Disease Epidemiology Section at the Minnesota Department of Health. His research interests include the epidemiology of foodborne and vector-borne diseases as well as immigrant and refugee health.

Dr. Bender is an assistant professor at the University of Minnesota, College of Veterinary Medicine, Division of Veterinary Public Health, and was the former state public health veterinarian at the Minnesota Department of Health. His research interests include food safety, zoonoses, and emerging diseases.

### References

1. World Health Organization. Malaria. <http://www.who.int/inf-fs/en/fact094.html> October 1998 (Accessed September 1999).
2. Ackerknecht EH. Malaria in the Upper Mississippi Valley: 1760-1900. Baltimore: Johns Hopkins Press; 1945.
3. Daggy RH, Muegge OJ, Riley WA. A preliminary survey of the anopheline mosquito fauna of southeastern Minnesota and adjacent Wisconsin areas. In: Melton LJ. Malaria in Minnesota: past, present, and future. Minn Med 1998;81:41-4.
4. Department of State, Department of Justice, and Department of Health and Human Services: U.S. refugee admissions for fiscal year 2000. <http://www.usinfo.state.gov/topical/global/refugees/fy2000.pdf> October 1999 (Accessed September 2000).
5. Kain KC, Harrington MA, Tennyson S, Keystone JS. Imported malaria: prospective analysis of problems in diagnosis and management. Clin Infect Dis 1998;27:142-9.
6. Snounou G, Viriyakosol S, Zhu XP, Jarra W, Pinheiro L, do Rosario VE, et al. High sensitivity of detection of human malaria parasites by the use of nested polymerase chain reaction. Mol Biochem Parasitol 1993;61:315-20.
7. Minnesota Department of Health: Refugee health screening in Minnesota: current status and recommendations. Disease Control Newsletter 1997;25:37-41.
