# Erratum Vol. 7 No. 6

**DOI:** 10.3201/eid0801.030101

**Published:** 2002-01

 In “The Changing Epidemiology of Malaria in Minnesota,” S.A. Seys and J.B. Bender, a number was inadvertently inserted into a sentence. On page 993, column 2, second full paragraph, the third sentence should read, “Among the patients who were born abroad, sites of malarial infection were Africa and Asia.”

 We regret any confusion this error may have caused.

